# P-1622. Decreasing Cellulitis Length of Stay with Implementation of Rapid Diagnostic Test

**DOI:** 10.1093/ofid/ofae631.1789

**Published:** 2025-01-29

**Authors:** Rishita Shah, Stephen Grohmann, Catherine Hart

**Affiliations:** Northwestern Lake Forest Hospital, Lake Forest, IL, Illinois; Northwestern Lake Forest Hospital (Lake Forest, IL), Chicago, Illinois; NORTHWESTERN MEDICINE, Lake Forest, Illinois

## Abstract

**Background:**

Skin and soft tissue infections (SSTIs) have been an increasing burden in the recent years. Rapid diagnostic tests can be useful to rule out *Staphylococcus aureus* as a pathogen. Northwestern Lake Forest Hospital (NLFH) did not have MRSA purulent wound PCR onsite and was sent out to another hospital. It was noticed that patients had longer length of stay (LOS) in our system compared to cohorts with patients diagnosed with cellulitis. We evaluated patients with cellulitis without major complications (DRG 603) and found an average LOS of 0.86. The goal was to reduce LOS of patients with cellulitis without major complications with the addition of a rapid diagnostic tool in our lab.

Length of Stay Run Chart

**Figure:**
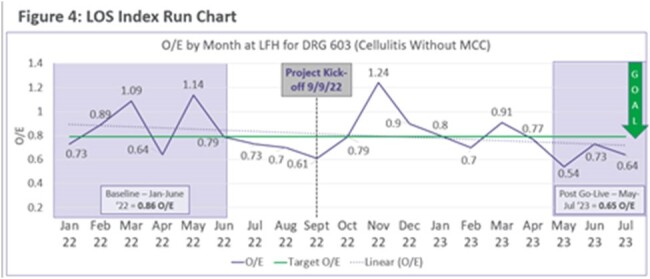

Shows LOS prior to project and after interventions

**Methods:**

NLFH was under-utilizing rapid diagnostic testing to quickly identify if *Staphylococcus aureus* was an organism for SSTI. We also analyzed turnaround times (TAT) of send out testing compared to on-site testing with regards to observed to expected LOS. We used a multi-modal and phased approach with education, upgrades to our electronic health record (EHR), creation of an updated SSTI Clinical Pathway, and implementation of onsite MRSA purulent wound PCR (Table 1). Our primary goal was to reduce LOS. Another goal was to reduce TAT of testing.

Interventions were applied January to June of 2022 and post intervention data was collected May – July 2023. Education was done to emphasize ordering in the ED. Providers were informed on EHR updates in Epic and best practice alerts were implemented as a reminder to place a picture in the EHR and order correct tests.

Summary of Interventions

**Figure:**
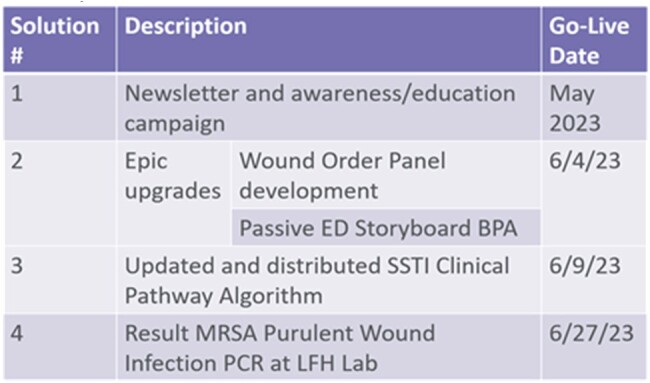

**Results:**

Baseline data was collected and observed over expected length of stay was 0.89. Among other NM hospitals across our system, we were in the 79^th^ percentile for cellulitis without major complications. TAT for testing results was also elevated in the pre-intervention timeframe to an average of 21 hours. We found a reduction in LOS with implementation of MRSA purulent wound PCR and education to 0.65. NLFH ranked in the 90^th^ percentile across our system during the post analysis period. When we evaluated TAT during our post intervention period, the time decreased to an average of 6 hours.

MRSA purulent wound PCT Turn Around Times

**Figure:**
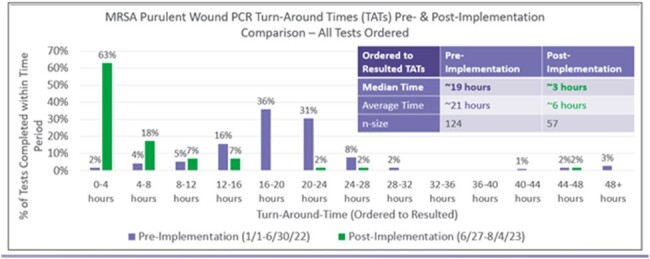

Shows pre and post interventions TAT for testing

**Conclusion:**

Implementation of a MRSA purulent wound PCR at NLFH helped improve TAT to identification of Staphylococcus aureus. Next steps would be to evaluate the impact on vancomycin utilization.

**Disclosures:**

**All Authors**: No reported disclosures

